# Virologic, Immunologic and Clinical Responses in Foreign-Born versus US-Born HIV-1 Infected Adults Initiating Antiretroviral Therapy: An Observational Cohort Study

**DOI:** 10.1371/journal.pone.0052336

**Published:** 2012-12-19

**Authors:** Deidra D. Parrish, Meridith Blevins, Samuel E. Stinnette, Peter F. Rebeiro, Bryan E. Shepherd, Timothy R. Sterling, Catherine C. McGowan, C. William Wester

**Affiliations:** 1 Vanderbilt University Institute for Global Health, Nashville, Tennessee, United States of America; 2 Vanderbilt University School of Medicine Dept of Biostatistics, Nashville, Tennessee, United States of America; 3 Vanderbilt University School of Medicine Division of Infectious Diseases, Nashville, Tennessee, United States of America; 4 Vanderbilt Comprehensive Care Clinic, Nashville, Tennessee, United States of America; 5 Harvard School of Public Health, Dept of Immunology and Infectious Diseases, Boston, Massachusetts, United States of America; Fundacion Huesped, Argentina

## Abstract

**Introduction:**

Mortality rates within the first year of combination antiretroviral therapy (cART) initiation are several-fold higher in resource-limited countries than in resource-replete settings. However studies in western countries examining virologic, immunologic and clinical responses after cART initiation in indigenous versus non-indigenous populations have shown mixed results. This study aimed to determine whether there is a difference in these outcomes in a United States setting between foreign-born and US-born patients.

**Methods:**

This retrospective observational cohort study of HIV-1 infected adults in one urban clinic in the United States compared virologic suppression, immune recovery and rates of AIDS defining events (ADEs) within the first year of cART using linear mixed effect models, log rank tests and Cox proportional hazard models. Data were analyzed for 94 foreign-born and 1242 US-born patients.

**Results:**

Foreign-born patients were younger (31.7 years versus 38.5 years), more often female (38.3% versus 27.1%), less often injection drug users (3.2% versus 9.5%) or men who have sex with men (19.0% versus 54.5%), and had higher loss to follow-up rates (14.9% versus 6.2%). No significant differences were detected between the groups in suppression of plasma HIV-1 RNA, CD4+ cell recovery or development of ADEs.

**Conclusions:**

During the first year on cART, virologic suppression, immune recovery and development of ADEs were comparable between foreign-born and US-born patients in care in a US clinic. Differential rates of loss to follow-up warrant further investigation in the foreign-born population.

## Introduction

Patient outcomes in HIV-infected adults initiating combination antiretroviral therapy (cART) have been shown to vary depending on setting. Notably, high rates of early mortality in the months after cART have been reported from several cohorts in resource limited countries, with early mortality rates several fold higher in lower-income countries than in high-income countries.[1]–[5] When examining different populations in the same setting, studies comparing virologic and immunologic outcomes have shown mixed results. Cohort studies from the United Kingdom and the Netherlands demonstrated poorer virologic response and higher rates of disease progression in non-indigenous patients receiving care in those countries. [6]–[8] However, analyses from Swiss and Spanish cohorts showed no such differences between native and non-native populations. [9], [10].

In the United States, foreign-born individuals make up approximately 13% of the population and accounted for approximately 16% of the HIV diagnoses between 2007 to 2010. [11] Results of epidemiologic studies from the US have also been mixed, with one finding that foreign-born patients were more likely to have AIDS at diagnosis while another showed no difference in concurrent HIV/AIDS diagnosis compared to US-born patients. [12], [13] No studies have compared outcomes of foreign-born versus US born patients on antiretroviral treatment. The aim of this study was to determine whether there was a difference in virologic, immunologic and clinical outcomes within one year of cART initiation between foreign-born and US-born HIV-1 infected adults receiving care in one urban outpatient HIV clinic in the United States. We hypothesized that patients from resource-limited regions of the world would experience poorer virologic suppression and immunologic recovery and would have higher rates of AIDS-defining events (ADEs).

## Methods

We performed a retrospective cohort study among persons in care at the Comprehensive Care Clinic, a Vanderbilt University-affiliated public outpatient HIV clinic in Nashville, Tennessee, USA. The Comprehensive Care Clinic accepts patients regardless of nationality, insurance status or immigration status, with more than 80% of the clinic patients utilizing services funded by the Ryan White Program, a government assistance program for HIV/AIDS care. The study population included all previously ART-naive patients initiating cART between January 1, 1998 and December 31, 2007 whose information had been compiled on a de-identified dataset. To estimate the association between birthplace and viral load suppression and CD4+ cell recovery, a repeated measures analysis was performed on the data using a linear mixed-effects model adjusted for sex, age and baseline CD4+ cell count, creating best fit curves of estimated expected CD4+ cell counts and plasma HIV-1 RNA levels. Time since cART initiation was included in the model using natural splines with three knots. Because the limits of detection for HIV-1 RNA assays changed over time (from <400 copies/ml to <48 copies/ml), we used multiple imputation to assign HIV-1 RNA values of <400 to be from 49 to 399 copies/mL; values <48 were assigned to be 49 copies/mL. The proportion of patients with suppressed viral load was estimated using the distribution of the predicted values of the linear mixed-effects model. Kaplan-Meier estimates were used to calculate hazard rates of death or developing an ADE during the year following cART initiation. A log rank test assessed for differences in rates by birthplace, and Cox proportional hazard models assessed the relationship between time to death or time to ADE and baseline variables. Missing values of baseline predictors were accounted for using single imputation techniques. Country of origin was determined by patient self-report. Patients without documented country of origin (n = 12) were assumed to be from the United States. Foreign-born patients from resource-replete settings (n = 2) were excluded. Patients were deemed lost to follow-up if they had less than one year of follow-up and were not reported as dead within one year of starting cART. Combined antiretroviral therapy was defined as (1) at least two nucleoside reverse-transcriptase inhibitors (NRTIs) in combination with at least one protease inhibitor (PI) and/or non-NRTI (NNRTI); (2) one NRTI and any PI and any NNRTI; or (3) at least 3 NRTIs. R software 2.15.1 (R Foundation for Statistical Computing, Vienna, Austria, available at: http://www.r-project.org) was used for all data analyses.

All patients consented to have their data stored in the clinic’s electronic medical database. The database was stripped of all protected health information before its use in research. Because we used this existing de-identified data set, the Vanderbilt University Institutional Review Board deemed the research exempt from review.

## Results and Discussion

1336 patients met inclusion criteria, consisting of 94 foreign-born and 1242 US-born patients (Table 1). Forty-nine of the 94 foreign-born patients (52.1%) were from Latin America, with Mexico the most common country of origin (35/94; 37.2%). A number of baseline characteristics were significantly different between foreign-born and US-born groups including age at cART initiation (median of 31.7 versus 38.5 years, respectively), female sex (38.3% versus 27.1%) and HIV risk factor, with foreign-born patients less likely to have had exposure via injection drug use (3.2% versus 9.5%, respectively) or male to male sexual contact (19.0% versus 54.5%, respectively). Foreign-born patients were more likely to be lost-to-follow-up during the first year (14.9% versus 6.2% in US-born).

**Table 1:** Patient Characteristics by Birth Location and Sex.

	Foreign-born women	Foreign-born men	All Foreign-born	US-born women	US-born men	All US-born	Combined	P-value
	(n = 36)	(n = 58)	(n = 94)	(n = 337)	(n = 905)	(n = 1242)	(n = 1336)	Foreign-born vsUS-born
Age at cART initiation (years)	28.3 (24.7, 34.8)	32.5 (29.4, 39.7)	31.7 (27.3, 38.6)	36.1 (28.6, 43.9)	39.2 (33.4, 44.8)	38.5 (32.1, 44.5)	38 (31.5, 44.3)	<0.001
BMI (kg/m^2^)	25.1 (23.8, 30.7)	23.1 (20.8, 27)	23.6 (22, 28.5)	26.5 (22.9, 33.5)	23.7 (21.1, 26.9)	24.4 (21.4, 28.2)	24.3 (21.4, 28.2)	0.772
Missing BMI, n(%)	13 (36.1%)	18 (31.0%)	31 (33.0%)	70 (20.8%)	190 (21.0%)	260 (20.9%)	291 (21.8%)	
Risk factor								<0.001
Heterosexual	21 (58.4%)	26 (44.8%)	47 (50%)	213 (63.2%)	147 (16.2)	360 (29%)	407 (30.5%)	
MSM	0 (0%)	11 (19%)	11 (11.7%)	0 (0%)	483 (53.3%)	483 (38.9%)	494 (37%)	
IDU	0 (0%)	3 (5.2%)	3 (3.2%)	40 (11.9%)	78 (8.6%)	118 (9.5%)	121 (9.1%)	
IDU/MSM	0 (0%)	0 (0%)	0 (0%)	0 (0%)	11 (1.2%)	11 (0.9%)	11 (0.8%)	
Other/Unknown	15 (41.7%)	18 (31%)	33 (35.1%)	84 (22.9%)	186 (20.6%)	270 (21.8%)	303 (22.7%)	
CD4 count (cells/µl)	286 (115.5, 364.5)	149 (36.8, 238.5)	195.5 (66.8, 298.5)	238 (91, 380.8)	179.5 (59, 294)	192 (63, 306.2)	192 (63, 306)	0.890
Missing CD4 count, n(%)	0 (0.0%)	0 (0.0%)	0 (0.0%)	21 (6.2%)	37 (4.1%)	58 (4.7%)	58 (4.3%)	
Hemoglobin (g/dl)	12.1 (11.3, 12.9)	13.6 (12.4, 14.6)	12.7 (11.4, 14)	12 (11.2, 12.9)	13.8 (12.3, 15.1)	13.2 (11.7, 14.7)	13.2 (11.7, 14.6)	0.142
Missing hemoglobin, n(%)	5 (13.9%)	18 (31.0%)	23 (24.5%)	75 (22.3%)	228 (25.2%)	303 (24.4%)	326 (24.4%)	
HIV-1 RNA level (log10)	4.3 (3.6, 5)	4.8 (4.3, 5.3)	4.6 (4.1, 5.3)	4.6 (4, 5.1)	4.9 (4.5, 5.5)	4.9 (4.4, 5.4)	4.8 (4.4, 5.4)	0.055
Missing HIV-1 RNA level, n(%)	0 (0.0%)	0 (0.0%)	0 (0.0%)	18 (5.3%)	32 (3.5%)	50 (4.0%)	50 (3.7%)	
One Year Mortality, n(%)								
Alive	32 (88.9%)	47 (81.0%)	79 (84.0%)	302 (89.6%)	810 (89.5%)	1112 (89.5%)	1191 (89.1%)	
Dead	0 (0.0%)	1 (1.7%)	1 (1.1%)	15 (4.5%)	38 (4.2%)	53 (4.3%)	54 (4.0%)	
Lost	4 (11.1%)	10 (17.2%)	14 (14.9%)	20 (5.9%)	57 (6.3%)	77 (6.2%)	91 (6.8%)	0.002
One Year ADE Incidence, n(%)								
No ADE	30 (83.3%)	40 (69.0%)	70 (74.5%)	284 (84.3%)	737 (81.4%)	1021 (82.2%)	1091 (81.7%)	
ADE	2 (5.6%)	9 (15.5%)	11 (11.7%)	35 (10.4%)	117 (12.9%)	152 (12.2%)	163 (12.2%)	
Lost	4 (11.1%)	9 (15.5%)	13 (13.8%)	18 (5.3%)	51 (5.6%)	69 (5.6%)	82 (6.1%)	0.005

To compare the distribution of patient characteristics by birthplace, chi-square tests and a two-sample rank sum test for continuous variables were used. Continuous variables are reported as medians (interquartile range).

**Figure 1:**
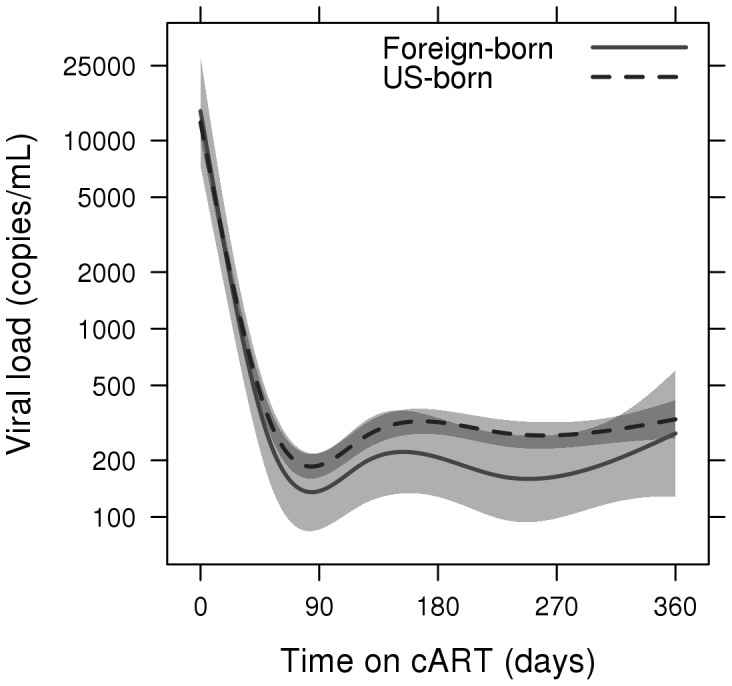
Viral load by time on combined antiretroviral therapy. Adjusted for sex, age and baseline CD4+ cell count.

**Figure 2:**
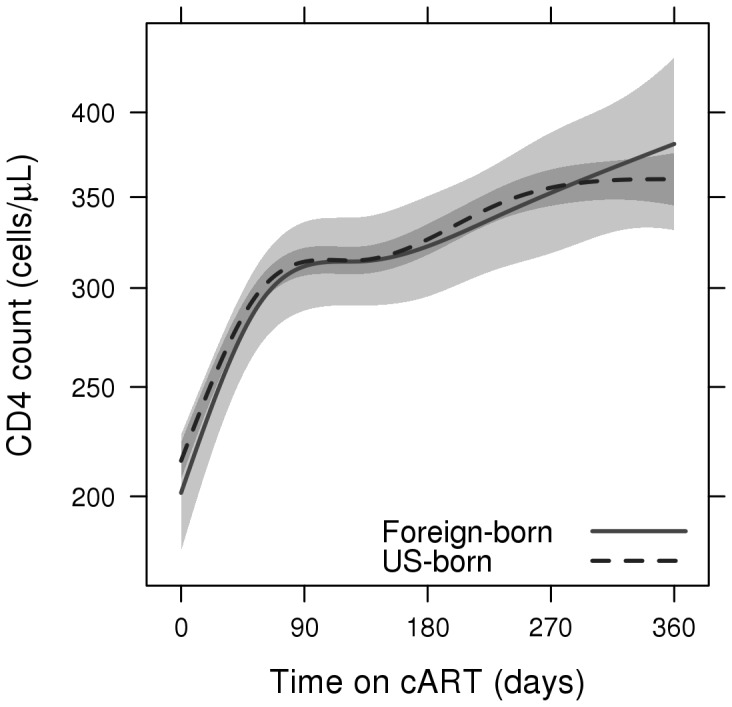
CD4+ cell count by time on combined antiretroviral therapy. Adjusted for sex, age and baseline CD4+ cell count.

Plasma viral load suppression and CD4+ cell recovery remained comparable in the two groups throughout the year (Figures 1 and 2), with overlapping confidence intervals in both the plasma HIV-1 RNA level and CD4+ cell count curves. At 90 days, 70.4% of foreign-born and 64.8% of US born patients had suppressed viral loads (plasma HIV-1 RNA <400 copies/ml). Estimated expected plasma HIV-1 RNA levels were 137 copies/ml (95% CI: 86–220) and 188 copies/ml (95% CI: 162–218) in foreign-born and US-born patients respectively while expected CD4+ cell counts were 312 cells/ul (95% CI: 288–336) and 314 cells/ul (95% CI: 307–322). At 360 days, 57.2% of foreign-born and 53.8% of US born patients had suppressed viral loads. Estimated expected HIV-1 RNA levels in foreign-born and US-born patients were 278 copies/ml (95% CI: 128–602) and 330 copies/ml (95% CI: 261–418), respectively, with CD4+ cell counts of 381 cells/ul (95% CI: 331–434) and 360 cells/ul (95% CI: 345–376). Rates of AIDS-defining events (ADE)s were similar in the two groups, with 11 foreign-born patients (11.7%) versus 152 US-born patients (12.2%) developing ADEs within the first year following cART initiation (aHR 1.04; 95% CI: 0.57–1.93). One year crude mortality rates were 1.2% and 4.7% in foreign-born and US-born patients respectively (aHR 0.26; 95%CI 0.04–1.87).

Those who were lost to follow-up were younger (35.2 vs. 38 years) and had slightly lower body mass index and CD4+ cell counts than those who were not lost (data not shown). To further characterize those lost to follow-up, we compared baseline attributes of the lost to follow-up group to patients who were alive and to patients who had died at the end of follow-up to determine which differences were statistically significant. Compared to the alive group, those lost to follow-up had a lower body mass index (23 versus 24.5 kg/m^2^, p = 0.02). Compared to the dead group, patients lost to follow-up were younger at cART initiation (35.2 versus 40.7 years, p<0.01), had higher CD4+ cell counts (median 168 versus 95 cells/ul, p<0.01) and higher hemoglobin levels (median 13.1 versus 11.3 g/dl, p<0.01). These differences, though statistically significant, may or may not be clinically significant.

Contrary to our hypothesis, virologic suppression and CD4+ cell count recovery were similar between foreign-born and US-born patients during the first year on cART. Likewise, foreign-born patients did not appear more likely to develop ADEs. Limitations of our study include the relatively small sample size and higher lost-to-follow-up rates in the foreign-born group, which could have affected our ADE results; this along with the low death rates, limits the reliability of the crude mortality analysis. We believe our analyses of plasma HIV-1 RNA level and CD4+ cell count were less affected by loss-to-follow-up because similar percentages of patients in both groups had viral load and CD4+ cell count data recorded at defined intervals during the year (data not shown). There were variables related to the patients’ HIV infection that were not available to the investigators, but may potentially have an effect on outcomes, such as country of HIV acquisition, HIV subtype and duration of infection. In addition, our data are from a single urban US site, so our findings may not be generalizable to other resource-replete settings.

Our cohort had a lower proportion of HIV in foreign-born individuals than the national average. This is likely due to the population distribution of foreign-born persons in the United States. Prosser et al noted the variations that exist in the distribution of foreign-born persons and patterns of infection at the state and regional levels. [11].

### Conclusions

The present study detected no significant differences in virologic suppression, CD4+ cell recovery or development of ADEs in the year after starting cART between foreign-born and US-born HIV-1 infected patients receiving care in one urban US setting. Given the higher loss to follow-up rates among foreign-born patients, a focus on identifying and addressing barriers to retention in care is warranted.
